# Graph embeddings on gene ontology annotations for protein–protein interaction prediction

**DOI:** 10.1186/s12859-020-03816-8

**Published:** 2020-12-16

**Authors:** Xiaoshi Zhong, Jagath C. Rajapakse

**Affiliations:** 1 School of Computer Science and Technology, Beijing Institute of Technology, Beijing, China; 2 School of Computer Science and Engineering, Nanyang Technological University, 50 Nanyang Avenue, Singapore, Singapore

**Keywords:** Graph embeddings, Vector representations, Gene Ontology annotations, Protein–protein interactions, Missing PPIs, Spurious PPIs

## Abstract

**Background:**

Protein–protein interaction (PPI) prediction is an important task towards the understanding of many bioinformatics functions and applications, such as predicting protein functions, gene-disease associations and disease-drug associations. However, many previous PPI prediction researches do not consider missing and spurious interactions inherent in PPI networks. To address these two issues, we define two corresponding tasks, namely missing PPI prediction and spurious PPI prediction, and propose a method that employs graph embeddings that learn vector representations from constructed Gene Ontology Annotation (GOA) graphs and then use embedded vectors to achieve the two tasks. Our method leverages on information from both term–term relations among GO terms and term-protein annotations between GO terms and proteins, and preserves properties of both local and global structural information of the GO annotation graph.

**Results:**

We compare our method with those methods that are based on information content (IC) and one method that is based on word embeddings, with experiments on three PPI datasets from STRING database. Experimental results demonstrate that our method is more effective than those compared methods.

**Conclusion:**

Our experimental results demonstrate the effectiveness of using graph embeddings to learn vector representations from undirected GOA graphs for our defined missing and spurious PPI tasks.

## Background

Protein–protein interactions (PPI) play an important role in understanding functional properties of proteins and their potentials as biomarkers. Predicting interactions between proteins is a crucial step in many bioinformatics applications such as identifying drug–target interactions [1, 2], construction of PPI networks (PPIN) [3–5], and detection of functional modules [6, 7]. The task aiming at predicting interactions between proteins is often termed as PPI prediction [8, 9].

PPI prediction is a well investigated problem in bioinformatics; for example, Struct2Net was used to integrate the structural information for PPI prediction [10, 11], PSOPIA leveraged on sequence information for PPI prediction [12], and several other research [9, 13–18]. However, these methods implicitly assume that known interactions between proteins are perfect and focus mainly on prediction task using existing PPIN that are incomplete and contain missing and spurious PPI, affecting their applications. A few existing PPI prediction methods have considered missing and spurious (i.e., erroneous) interactions of PPIN.

To address issues of incompleteness and spuriousness, we define two specific tasks on PPIN: (i) missing PPI prediction and (ii) spurious PPI prediction. For the missing PPI prediction, we treat a real PPI dataset as the ground-truth PPI dataset, remove PPIs randomly, and attempt to predict them as missing PPI. The goal of missing PPI prediction is to see whether we could correctly predict the missing PPI. For the spurious PPI prediction, we add some PPIs to the ground-truth PPI dataset, treat them as spurious PPIs, and try to predict them. The goal of spurious PPI prediction is to see the extent of correctly predicting the spurious PPIs.

The majority of PPI prediction methods leverage on the information from Gene Ontology (GO) that provides a set of structured and controlled vocabularies (or terms) describing gene products and molecular properties [19]. Proteins are generally annotated by a set of GO terms [20, 21]. For example, the protein “Q9NZJ4” is annotated by the following GO terms: “GO:0003674”, “GO:0005524”, “GO:0005575”, “GO:0006457”, “GO:0006464”, and “GO:0031072”. Based GO term-protein annotations, many research have employed information content (IC) of GO terms [22–25] to compute similarity between two proteins in order to predict PPI. These methods have succeeded in the development of protein-related tasks, including PPI prediction [26–33]. Despite their success, IC-based methods have been unable to fully capture functional properties of proteins and structural properties of PPIN.

Recently, several researchers have proposed word embeddings (e.g., word2vec [34] and GloVe [35]), which have been developed in the area of natural language processing, to learn vector representations of GO terms and proteins and then used learned vectors for the PPI prediction [36–39]. These methods mainly use the word2vec model [34] to learn vectors for each word from the corpus derived from descriptive axioms of GO terms and proteins; the descriptive axiom of a GO term is its textual description, for example, the descriptive axiom of the GO term “GO:0036388” is “pre-replicative complex assembly.” Then, the learned word vectors are combined into vectors of GO terms and proteins, according to the words in the descriptive axioms of GO terms and proteins. Finally, the vectors of proteins are used to predict the protein interactions. We have earlier proposed GO2Vec [39] that convert the GO graph into a vector space to represent genes for predicting their similarity.

Extending our previous work [39, 40], in this paper, we propose to derive graph embeddings to transform GO annotation (GOA) graph into their vector representations in order to predict missing and spurious PPI. Specifically, using GOA, our method first combines term–term relations between GO terms and term-protein annotations between GO terms and proteins, and then constructs an undirected and unweighted graph; this constructed graph is called the GOA graph. Thereafter, node2vec model [41], one of graph embedding models, is applied on the GOA graphs to transform the nodes (including GO terms and proteins) into their vector representations. By taking GOA for embeddings instead of GO, we take information on how gene functions are related in individual proteins. Finally, learned vectors of GO terms and proteins with the cosine distance and the modified Hausdorff distance [42] measures are used to predict missing and spurious PPI.

Our method can capture the structural information connecting the nodes in the entire GOA graph. On one hand, when compared with structure-based IC methods that mainly consider the nearest common ancestors of two nodes, graph embeddings take into account the information from every path between two nodes. Graph embeddings therefore can fully portray the relationship of two nodes in the entire graph. On the other hand, when compared with the corpus-based methods, including the traditional IC based methods and word embedding based methods, graph embeddings can employ the expert knowledge (e.g., term–term relations and term-protein annotations) stored in the graphical structure. In our experiments, we used the node2vec model [41] as the representative of graph embedding techniques. The node2vec model adopts a strategy of random walk over an undirected graph to sample neighborhood nodes for a given node and preserves both neighborhood properties and structural features.

To evaluate the quality of our proposed methods in addressing the issues of missing and spurious PPIs, we conducted experiments on three PPI datasets (i.e., HUMAN, MOUSE, and YEAST) from the STRING database [43], considering three GO categories, i.e., Biological Process (BP), Cellular Component (CC), and Molecular Function (MF), with the GO annotations collected from the UniProt database [44]. We compared our methods with representative IC-based methods including Resnik [24], Lin [23], Jang and Conrath [22], simGIC [25], and simUI [45], and a recent corpus-based vector representation method Onto2Vec [36]. Experimental results demonstrate the effectiveness of our methods over existing methods in both missing and spurious PPI predictions. We conclude that combining term–term relations between GO terms and term-protein annotations between GO terms and proteins by using GOA graph embeddings accurately represents gene in the Euclidean space reflecting their functional properties.

**Fig. 1:**
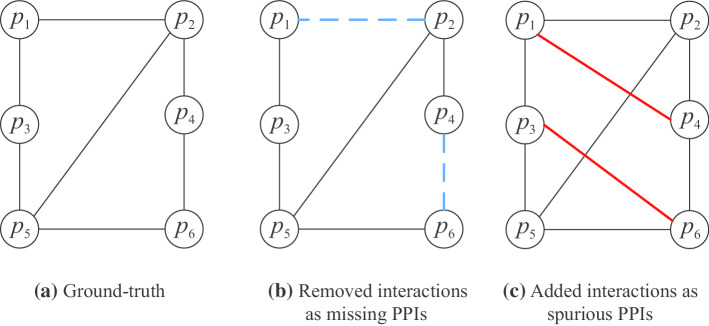
Illustration of missing and spurious PPI predictions. **a** The ground-truth PPI graph derived from a real-world PPI dataset where nodes are the proteins and edges represent PPI. **b** A derived PPI graph, with two PPIs (indicated by the blue dashed edges) removed from **a**, and is used for missing PPI prediction where the blue dashed edges are missing PPIs. **c** A derived PPI graph, with two PPIs (indicated by the red bold edges) added to **a** and is used for spurious PPI prediction where the red bold edges are spurious PPIs

## Results

### Preliminary task definitions

In this paper, we consider two kinds of PPI prediction tasks, namely missing PPI prediction and spurious PPI prediction. Figure 1 illustrates the constructions of missing PPIs and spurious PPIs. Graph (a) is given by a real-world PPI dataset and is treated as the ground-truth PPI graph. Graph (b) is derived from Graph (a) by removing some PPIs and these removed PPIs are treated as missing PPIs. Graph (c) is also derived from Graph (a), but instead of removing PPIs, some PPIs are added to Graph (a) and these added PPIs are treated as spurious PPIs.

#### Missing PPI prediction

Given a ground-truth PPI graph with some PPI removed (e.g., Graph (b)), the goal of missing PPI prediction is to predict whether these removed PPIs are missing PPI.

#### Spurious PPI prediction

Given a ground-truth PPI graph with some PPIs added (e.g., Graph (c)), the goal of spurious PPI prediction is to predict whether these added PPIs are spurious PPI.

### Experimental results

We conducted experiments on missing PPI prediction and spurious PPI prediction tasks and evaluated the performance in comparison with representative IC-based methods including Resnik [24], Lin [23], Jang and Conrath [22], simGIC [25], and simUI [45]), and recent corpus-based vector representation method Onto2Vec [36] on three PPI datasets (HUMAN, MOUSE, and YEAST) from the STRING database [43].

**Table 1:** AUC-ROC values for *missing* PPI prediction

GO	Model	Human	Mouse	Yeast
BP	Resnik	0.8257	0.8154	0.8224
	Lin	0.8065	0.7831	0.7752
	Jang and Conrath	0.7973	0.7694	0.7610
	simGIC	0.8147	0.7775	0.7914
	Onto2Vec	0.8458	0.8316	0.8416
	GOA2Vec (cos)	0.8513	0.8419	0.8674
	GOA2Vec (mhd)	0.8676	0.8527	0.8718
	GOA2Vec (svm)	*0*.*8814*	*0*.*8728*	*0*.*8889*
CC	Resnik	0.7776	0.7826	0.7916
	Lin	0.7165	0.7251	0.7435
	Jang and Conrath	0.7134	0.7295	0.7201
	simGIC	0.7658	0.7761	0.7715
	Onto2Vec	0.7984	0.8016	0.8068
	GOA2Vec (cos)	0.8027	0.8196	0.8035
	GOA2Vec (mhd)	0.8237	0.8349	0.8146
	GOA2Vec (svm)	*0*.*8396*	*0*.*8517*	*0*.*8358*
MF	Resnik	0.7934	0.7815	0.7916
	Lin	0.7335	0.7428	0.7432
	Jang and Conrath	0.7129	0.7349	0.7216
	simGIC	0.7618	0.7796	0.7794
	Onto2Vec	0.7953	0.7954	0.8059
	GOA2Vec (cos)	0.8115	0.8145	0.8243
	GOA2Vec (mhd)	0.8223	0.8316	0.8253
	GOA2Vec (svm)	*0*.*8397*	*0*.*8608*	*0*.*8411*

GO refers to ontology type used

**Table 2:** AUC-ROC values for *spurious* PPI prediction

Onto	Model	Human	Mouse	Yeast
BP	Resnik	0.8243	0.7935	0.7917
	Lin	0.7758	0.7514	0.7572
	Jang and Conrath	0.7494	0.7427	0.7348
	simGIC	0.7965	0.7638	0.7823
	Onto2Vec	0.8426	0.8167	0.8051
	GOA2Vec (cos)	0.8613	0.8207	0.8324
	GOA2Vec (mhd)	0.8725	0.8439	0.8467
	GOA2Vec (svm)	*0*.*8809*	*0*.*8613*	*0*.*8654*
CC	Resnik	0.7827	0.7758	0.8016
	Lin	0.7334	0.7364	0.7452
	Jang and Conrath	0.7157	0.7296	0.7291
	simGIC	0.7608	0.7710	0.7776
	Onto2Vec	0.8016	0.7913	0.7935
	GOA2Vec (cos)	0.8191	0.8142	0.8117
	GOA2Vec (mhd)	0.8360	0.8207	0.8254
	GOA2Vec (svm)	*0*.*8415*	*0*.*8427*	*0*.*8394*
MF	Resnik	0.7903	0.7817	0.7834
	Lin	0.7317	0.7298	0.7265
	Jang and Conrath	0.7186	0.7215	0.7184
	simGIC	0.7636	0.7716	0.7716
	Onto2Vec	0.8137	0.7903	0.8216
	GOA2Vec (cos)	0.8116	0.8134	0.8177
	GOA2Vec (mhd)	0.8209	0.8275	0.8194
	GOA2Vec (svm)	*0*.*8367*	*0*.*8416*	*0*.*8385*

GO refers to the ontology used

Table 1 reports overall performance of our proposed methods and existing methods for missing PPI prediction task. Table 2 reports overall performance of our models and existing methods for spurious PPI prediction. For each PPI dataset, different GO categories were used and best values are highlighted in italics.

#### Missing PPI prediction

As seen from Table 1, cosine distance (cos), modified Hausdorff distance (mhd), and Support Vector Machines (svm) achieved the best results on the missing PPI prediction compared to IC-based methods and corpus-based vector representation method on all the three PPI datasets. This indicates that graph embeddings can capture structural information from GOA graphs and functional properties of proteins effectively, which is useful for many applications including predicting the missing PPI.

Particularly, our proposed methods significantly outperform the traditional IC-based methods; the possible reason is that the IC-based methods consider only the information from the partial or local structure of a graph while GOA2Vec(cos), GOA2Vec(mhd), and GOA2Vec(svm) take into account the information from both the local and global structure of the GOA graphs, which incorporates the knowledge of both term–term relations between GO terms and term-protein annotations between GO terms and proteins. GOA2Vec(cos), GOA2Vec(mhd), and GOA2Vec(svm) on GOA embeddings also outperform the corpus-based vector representation method Onto2Vec. The may be due to the reason that GO and GOA represent more domain knowledge about genes, proteins, and their functionalities, than those represented by existing document composes.

Let us compare the performances of GOA2Vec(cos), GOA2Vec(mhd), and GOA2Vec(svm) classifications. GOA2Vec(svm) achieved better performance than GOA2Vec(cos) and GOA2Vec(mhd). The possible reason is that svm may have treated the problem as a binary classification, leveraging on the classification based on the largest margin between support vectors. Our experimental results also justify the usefulness of the functional annotation relationships between GO terms and proteins.

#### Spurious PPI prediction

As seen from Table 2, GOA2Vec(cos), GOA2Vec(mhd), and GOA2Vec(svm) outperformed both the IC-based methods and the corpus-based vector representation method on almost all the datasets except on the YEAST PPI dataset using the MF ontology. Similar to the performance on missing PPI prediction, this indicates again that graph embeddings can capture useful information from the structure of GOA graphs for the spurious PPI prediction, and that both the learned vectors of proteins and the ones of GO terms are effective for the spurious PPI prediction. In addition, GOA2Vec(svm) performed better than GOA2Vec(cos) and GOA2Vec(mhd) on spurious PPI prediction. This justifies again importance of considering the relationships between GO terms and proteins (term-protein annotations) in representing the proteins.

**Table 3:** AUC-ROC values between our method between using undirected graphs and using directed graphs for *missing* PPI prediction

GO	Model	Human	Mouse	Yeast
BP	svm	*0*.*8814*	*0*.*8728*	*0*.*8889*
	mhd	0.8676	0.8527	0.8718
	cos	0.8513	0.8419	0.8674
	d_svm	0.8354	0.8167	0.8279
	d_mhd	0.8134	0.8038	0.8295
	d_cos	0.8027	0.7924	0.8246
CC	svm	*0*.*8396*	*0*.*8517*	*0*.*8358*
	mhd	0.8237	0.8349	0.8146
	cos	0.8027	0.8196	0.8035
	d_svm	0.7968	0.8102	0.7991
	d_mhd	0.7837	0.8001	0.7766
	d_cos	0.7712	0.7931	0.7613
MF	svm	*0*.*8397*	*0*.*8608*	*0*.*8411*
	mhd	0.8223	0.8316	0.8253
	cos	0.8115	0.8145	0.8243
	d_svm	0.7954	0.8196	0.8007
	d_mhd	0.7884	0.7765	0.7835
	d_cos	0.7716	0.7664	0.7769

“d” stands for directed graph

**Table 4:** AUC-ROC values between different methods between using undirected graphs and using directed graphs for *spurious* PPI prediction

GO	Model	Human	Mouse	Yeast
BP	svm	*0*.*8809*	*0*.*8613*	*0*.*8654*
	mhd	0.8725	0.8439	0.8467
	cos	0.8613	0.8207	0.8324
	d_svm	0.8369	0.8194	0.8207
	d_mhd	0.8203	0.8034	0.8101
	d_cos	0.8167	0.7908	0.7964
CC	svm	*0*.*8415*	*0*.*8427*	*0*.*8394*
	mhd	0.8360	0.8207	0.8254
	cos	0.8191	0.8142	0.8117
	d_svm	0.8127	0.7961	0.7985
	d_mhd	0.8002	0.7834	0.7749
	d_cos	0.7763	0.7771	0.7746
MF	svm	*0*.*8367*	*0*.*8416*	*0*.*8385*
	mhd	0.8209	0.8275	0.8194
	cos	0.8116	0.8134	0.8177
	d_svm	0.7912	0.8027	0.7823
	d_mhd	0.7768	0.7824	0.7658
	d_cos	0.7834	0.7739	0.7549

“d” stands for directed graph

## Discussion

We find that using undirected graphs achieves better performance than using directed graphs does in this task. Tables 3 and 4 report comparisons between our proposed methods using undirected graphs and the ones using directed graphs for the missing and spurious PPI predictions. We can see that the methods that use undirected graphs perform much better than the corresponding methods that use directed graphs. The possible reason is that the node2vec model we use in this paper adopts a strategy of random walk over an undirected graph to sample neighborhood nodes for a given node and this strategy works better on undirected graphs than on directed graphs.

## Conclusions

In this paper, we employ graph embeddings to project Gene Ontology annotation graphs into vectors so as to predict the protein–protein interactions. We evaluate our method against traditional IC-based methods and a recent corpus-based word embedding method in the tasks of missing and spurious PPI predictions. Experimental results justify the effectiveness of our method to learn vectors from GOA graphs and the usefulness of the information of GO annotations for PPI predictions.

**Fig. 2:**
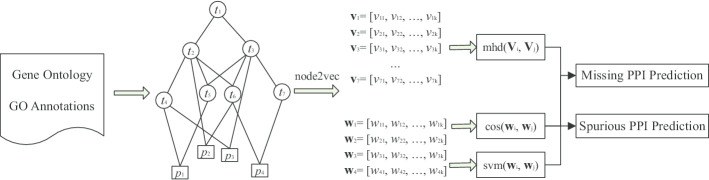
Steps involved in our method of PPI prediction. Firstly, GO and GOA are combined together to construct an undirected and unweighted GOA graph. The node2vec model is then applied on the GOA graph to transform the nodes to their vector representations. Finally, the learned vectors are used for the tasks of missing and spurious PPI predictions. *t*_*i*_ denotes a GO term and ${\mathbf {v}}_i = (v_{ij} )$ denotes its *k*-dimensional vector, $p_m$ denotes a protein *m* and ${\mathbf {w}}_m = ( w_{mn} )$ denotes its *k*-dimensional vector representing protein. ${\mathbf {V}}_m$ denote a set of vectors of GO terms that annotate the protein

## Methods

Figure 2 illustrates our method of missing and spurious PPI predictions, which consists of three components: (1) GOA graph construction, (2) transformation of GOA graph to vector representations, and (3) prediction of missing and spurious PPI.

### GOA graph construction

A GOA graph (or GO annotation graph) is an undirected and unweighted (or binary) graph, constructed from the GO and GOA. Specifically, we combine term–term relations between GO terms and term-protein annotations between GO terms and proteins together to form an undirected and unweighted graph where the nodes include both the GO terms and proteins, and the edges include both term–term relations and term-protein annotations.

Although GO is a directed acyclic graph (DAG) and transforming directed edges to undirected edges might result in a loss of some information, we found that graph embeddings working on undirected graphs achieved better performance than utilizing them on directed graphs. That is probably because the node2vec model we used adopts a strategy of random walks to sample neighborhood nodes, and such strategy works better on undirected graphs than on directed graphs. Therefore, in this paper, we constructed the GOA graph as an undirected graph by simply setting directed edges as undirected edges.

### GOA graph to vector representations

There are several graph embedding models that can be used to transform a graph to a vector space such as DeepWalk [46], LINE [47], and node2vec [41]. In our experiments, we found that the node2vec model works better in our datasets than other models and therefore node2vec was used to convert GOA graph into the Euclidean space. To make our paper self-contained, in what follows, we briefly introduce the node2vec model.

#### The node2vec model

Let (*N*, *E*) denote a graph, in which *N* indicates the set of nodes and $E \subseteq (N \times N)$ indicates the set of edges. The primary goal of node2vec is to learn a projecting function $f: N \rightarrow {\mathbb {R}}^k$ and transform these nodes to a set of vector representations in the space ${\mathbb {R}}^k$, where *k* indicates the dimensions of that space. *f* can be denoted by a matrix with the size $|N| \times k$. For a node $n \in N$, $N_b(n) \subset N$ indicates the set of *n*’s neighbourhood nodes, which are generated via a sampling method.

The node2vec model tries to optimize the log-probability of a set of observed neighborhood $N_b(n)$ for the node *n*, conditioned on its vector representation; this optimization problem is defined by Eq. (1).

1 $$\begin{aligned} \max _{f} \sum _{n \in N} \log P(N_b(n)|f(n)) \end{aligned}$$

To resolve this optimization problem, node2vec assumes conditional independence and symmetry in the feature space.

The conditional independence assumes that given the vector representation of a node *n*, the likelihood of observing a neighborhood node ${n}'$ does not depend on any other observed neighborhood node. This assumption is denoted by Eq. (2).

2 $$\begin{aligned} P(N_b(n)|f(n)) = \prod _{{n}' \in N_b(n)} P({n}'|f(n)) \end{aligned}$$

The symmetry in feature space assumes that the source node *n* and its neighborhood node ${n}'$ share a symmetric impact on each other in the feature space. This assumption is denoted by Eq. (3).

3 $$\begin{aligned} P({n}'|f(n)) = \frac{\exp (f({n}') \cdot f(n))}{\sum _{n'' \in N} \exp (f(n'') \cdot f(n))} \end{aligned}$$

Given these two assumptions, Eq. (1) is transformed to Eq. (4):

4 $$\begin{aligned} \mathop {\max }\nolimits _{f} \mathop {\sum }\nolimits _{n \in N} \left( \mathop {\sum }\nolimits _{{n}' \in N_b(n)} f({n}') \cdot f(n) - \mathop {\sum }\nolimits _{n'' \in N} \exp (f(n'') \cdot f(n))\right) \end{aligned}$$

For a source node *n*, node2vec simulates a random walk of the length *l*. Let $c_i$ represent the *i*-th node in the walk and start with $c_0=t$. The node $c_i$ is simulated by the following strategy:

5 $$\begin{aligned} P(c_i=x|c_{i-1}=n)={\left\{ \begin{array}{ll} \frac{\pi _{nx}}{Z} &{} \quad {\text {if}} \quad (n,x) \in E \\ 0 &{} \quad {\text {otherwise}} \end{array}\right. } \end{aligned}$$

where $\pi _{nx}$ denotes the transition probability between the nodes *n* and *x*; *Z* denotes a normalizing constant. For more details about the node2vec model, please refer to its original paper [41].

### Missing and spurious PPI predictions

After applying the node2vec model on the GOA graph for transformation, we get the vector representations for the GO terms and proteins. Specifically, each of GO terms and proteins is denoted by a *k*-dimensional vector. There are two ways to use these learned vectors to predict missing and spurious PPIs. One is to directly use these learned vectors of proteins; the other way is to use these learned vectors of GO terms.

#### Using learned vectors of proteins

Let ${\mathbf {w}}_s$ and ${\mathbf {w}}_t$ represent the learned vectors of protein $p_s$ and $p_t$. The similarity between two proteins $sim(p_s, p_t)$ can be calculated by the cosine distance $cos({\mathbf {w}}_s, {\mathbf {w}}_t)$ of their vector representations ${\mathbf {w}}_s$ and ${\mathbf {w}}_t$, defined by Eq. (6).

6 $$\begin{aligned} sim(p_s, p_t)=cos({\mathbf{w}}_s, {\mathbf{w}}_t)=\frac{{{\mathbf{w}}_s \cdot {\mathbf{w}}_t}}{\|{\mathbf{w}}_s\|\|{\mathbf{w}}_t\|} \end{aligned}$$

Besides the cosine distance, we also apply a support vector machine (SVM) on the learned vectors of proteins to train a classifier and treat the protein–protein interaction prediction as a binary classification problem. The two vectors ${\mathbf {w}}_s$ and ${\mathbf {w}}_t$ are used as input for the SVM classifier to classify the input to either 0 or 1 class, indicating presence or absence of an interaction. This method is denoted by $svm({\mathbf {w}}_s, {\mathbf {w}}_t)$ or simply svm.

#### Using learned vectors of GO terms

Since a protein is annotated by one or more GO terms, the protein *p* can be viewed as a set of its annotated GO terms. Let $N_s$ and $N_t$ represent the set of GO terms that annotate protein $p_s$ and $p_t$, respectively. To calculate the similarity between proteins $p_s$ and $p_t$, we can compute the similarity between their sets of GO terms, i.e., $N_s$ and $N_t$. Because a set of GO terms can be denoted by a set of its corresponding vectors, the similarity between two proteins can be calculated by the distance of these two sets of vectors. Let ${\mathbf {V}}_s$ represent the set of vectors corresponding to $N_s$, and let ${\mathbf {V}}_s$ represent the set of vectors that correspond to $N_t$. The similarity between two proteins $sim(p_s, p_t)$ can be derived from the similarity between two sets of vectors $sim(N_s, N_t)$, given by the distance between their corresponding sets of vectors $dist({\mathbf {V}}_s, {\mathbf {V}}_t)$:

7 $$\begin{aligned} sim(p_s, p_t)=sim(N_s, N_t)=dist({\mathbf {V}}_s, {\mathbf {V}}_t) \end{aligned}$$

There exists several ways to calculate the distance or similarity between two sets of vectors [28, 48]. In our experiments, we found that the modified Hausdorff distance [42] performed better than the simple linear combination of vectors. In this paper, therefore, we used the modified Hausdorff distance to calculate the distance between two sets of vectors for the similarity between two proteins.

For two data points in the Euclidean space, suppose that *dist* denotes the distance of the two data points in that space. A small *dist* indicates that the two data points are close. After GO terms are transformed into vectors, the $dist({\mathbf {v}}_i, {\mathbf {v}}_j)$ score indicates the spatial relationship between their corresponding GO terms $n_i$ and $n_j$. In our experiments, $dist({\mathbf {v}}_i, {\mathbf {v}}_j)$ is simply defined by the cosine distance. We used a variant of the modified Hausdorff distance [42] to calculate the distance between two sets of vectors for the similarity between two GO terms. Specifically, the modified Hausdorff distance is defined by Eq. (8) and it is denoted by $mhd({\mathbf {V}}_s, {\mathbf {V}}_t)$ in our research.

8 $$\begin{aligned} \min \left\{ \frac{1}{|{\mathbf {V}}_s|}\sum \nolimits _{{\mathbf {v}}_s\in {\mathbf {V}}_s}\max \nolimits _{{\mathbf {v}}_t\in {\mathbf {V}}_t}cos({\mathbf {v}}_s, {\mathbf {v}}_t), \frac{1}{|{\mathbf {V}}_t|}\sum \nolimits _{{\mathbf {v}}_t\in {\mathbf {V}}_t}\max \nolimits _{{\mathbf {v}}_s\in {\mathbf {V}}_s}cos({\mathbf {v}}_s, {\mathbf {v}}_t) \right\} \end{aligned}$$

where $|{\mathbf {V}}_s|$ represents the number of vectors in ${\mathbf {V}}_s$.

### Datasets

In this paper, we use three types of datasets: Gene Ontology, Gene Ontology Annotations, and Protein–Protein Interaction Network.

Gene Ontology: The Gene Ontology [19] contains three categories of ontologies that are independent of each other: BP, CC, and MF. The BP ontology contains those GO terms that depict a variety of events in biological processes. The CC ontology contains those GO terms that depict molecular events in cell components. The MF ontology contains those GO terms that depict chemical reactions, such as catalytic activity and receptor binding. These GO terms have been employed to interpret biomedical experiments (e.g., genetic interactions and biological pathways) and annotate biomedical entities (e.g., genes and proteins). Table 5 summarizes the statistics of the three categories of ontologies.

**Table 5:** Statistics of the three categories of ontologies

Ontology	#GO terms	#Edges
BP	30,705	71,530
CC	4380	7523
MF	12,127	13,658

“#GO Terms” indicates the number of GO terms and “#Edges” indicates the number of edges

Gene Ontology Annotations: GO annotations are statements about the functions of particular genes or proteins, and capture how a gene or protein functions at the molecular level, and what biological processes it is associated with. Generally, a protein is annotated by one or more GO terms. For example, the protein “Q9NZJ4” is annotated by the GO terms “GO:0003674”, “GO:0005524”, “GO:0005575”, “GO:0006457”, “GO:0006464”, and “GO:0031072”. We mapped the proteins to the UniProt^1^ database [44] to obtain the GO annotations, and we used the version of none Inferred from Electronic Annotation (no-IEA).

Protein–Protein Interaction Network: From the STRING database [43], we downloaded three kinds of PPI datasets (v11.0 version): HUMAN (Homo sapiens), MOUSE (Mus musculus), and YEAST (Saccharomyces cerevisiae). The HUMAN dataset contains 9677 proteins and 11,759,455 interactions, the MOUSE dataset contains 20,269 proteins and 8,780,518 interactions, and the YEAST dataset contains 3287 proteins and 1,845,966 interactions. We mapped the proteins to the UniProt database and filter out those proteins that could not be found in the UniProt database; we also discarded those interactions involving the filtered proteins. After filtering, the HUMAN dataset remains 6966 proteins and 1,784,108 interactions, the MOUSE dataset remains 16,105 proteins and 7,515,864 interactions, and the YEAST dataset remains 2851 proteins and 456,936 interactions. The remaining proteins and interactions in the three datasets were treated as their ground-truth PPI graphs.

**Table 6:** Statistics of the ground-truth PPI datasets as well as removed PPIs (“Re-PPI”) and added PPIs (“Ad-PPI”)

Dataset	#Protein	#PPI	#Re-PPI	#Ad-PPI
HUMAN	6966	1,784,108	500,000	500,000
MOUSE	16,105	7,515,864	500,000	500,000
YEAST	2851	456,936	100,000	100,000

We randomly sampled 500,000 HUMAN interactions, 500,000 MOUSE interactions, and 100,000 YEAST interactions from the ground-truth PPI graphs, and removed these sampled interactions from the ground-truth PPI graphs and treated them as missing PPIs. This kind of derived datasets is used for the missing PPI prediction.

From the ground-truth PPI datasets, we randomly sampled the same number of pairs of proteins (i.e., 500,000 interactions for HUMAN proteins, 500,000 interactions for MOUSE proteins, and 100,000 interactions for YEAST proteins), between which there are no interactions, and added them to the ground-truth PPI datasets. These added interactions were treated as spurious PPIs, and this kind of derived datasets is used for the spurious PPI prediction.

Table 6 summarizes the statistics of the proteins and interactions of the ground-truth PPI graphs, as well as the number of the removed PPIs and the added PPIs.

### Implementation details

We implemented several versions of our method in both ways that are described in Eqs. (6) and (8). The version that uses the learned vectors of proteins with cosine distance [Eq. (6)] is denoted by “cos”. The version that uses the learned vectors of GO terms with modified Hausdorff distance [Eq. (8)] is denoted by “mhd”. The version that uses the support vector machine to train a classifier is denoted by “svm”, and we use the version implemented in scikit-learn.

To investigate the effect of using undirected graphs, we also implemented three versions of GOA2Vec working on directed graphs. Their corresponding versions are denoted by “d_cos”, “d_mhd”, and “d_svm”, where “d” indicates using **d**irected graphs. Except using directed graphs, “d_cos” is the same as “cos”, “d_mhd” is the same as “mhd”, and “d_svm” is the same as “svm”.

For the node2vec model, we used its code^2^ on our datasets with trying different parameters and mainly reported the best results. The parameters that help us get the best results include: 150 dimensions, 10 walks per node, 80-length per walk and 20 walks per node, unweighted and undirected edges.

### Existing methods

Our method was compared with existing methods including the representative information content-based methods, namely Resnik [24], Lin [23], Jang and Conrath [22], simGIC [25], and simUI [45], and the corpus-based vector representation method Onto2Vec [36].

Resnik’s similarity is mainly based on the IC of a given node in an ontology. The IC of a node *n* is calculated by the negative log-likelihood, given by Eq. (9).

9 $$\begin{aligned} IC(n)=-\,\log p(n) \end{aligned}$$

where *p*(*n*) represents the probability of the node *n* over the whole nodes. Given this IC information, Resnik similarity is calculated by

10 $$\begin{aligned} sim_{Resnik}(n_1,n_2)=-\,\log p(n_m) \end{aligned}$$

where $n_m$ denotes the most informative common ancestor of $n_1$ and $n_2$ in that ontology.

Lin’s similarity [23] is calculated by

11 $$\begin{aligned} sim_{Lin}(n_1,n_2)=\frac{2 * \log p(n_m)}{\log p(n_1) + \log p(n_2)} \end{aligned}$$

Jang and Conrath’s similarity [22] is calculated by

12 $$\begin{aligned} sim_{J{ \& }C}(n_1, n_2)=2*\log p(n_m) - \log p(n_1) - \log p(n_2) \end{aligned}$$

simGIC similarity [25] and simUI similarity [45] calculate the similarity among proteins. Let $N_1$ and $N_2$ represent the set of GO terms that annotate the proteins $p_1$ and $p_2$, respectively. simGIC similarity is calculated by the Jaccard index, given by Eq. (13), while simUI similarity is calculated by the universal index, given by Eq. (14).

13 $$\begin{aligned} fun_{GIC}(p_1,p_2)&= \frac{\sum _{n \in N_1 \cap N_2} IC(n)}{\sum _{n \in N_1 \cup N_2} IC(n)} \end{aligned}$$

14 $$\begin{aligned} fun_{UI}(p_1,p_2)&= \frac{\sum _{n \in N_1 \cap N_2} IC(n)}{\max \{\sum _{n \in N_1} IC(n), \sum _{n \in N_2} IC(n)\}} \end{aligned}$$

There are three main kinds of methods that combine for Resnik’s, Lin’s, and Jang and Conrath’s similarities: average (AVG), maximum (MAX), and best-match average (BMA). These three combination methods are defined by Eqs. (15), (16), and (17), respectively.

15 $$\begin{aligned} fun_{AVG}(p_1,p_2)&= \frac{1}{|N_1||N_2|}\sum _{n_1 \in N_1, n_2 \in N_2} IC(\{n_1, n_2\}) \end{aligned}$$

16 $$\begin{aligned} fun_{MAX}(p_1,p_2)&= \max \{IC(\{n_1, n_2\})|n_1\in N_1, n_2\in N_2\} \end{aligned}$$

17 $$\begin{aligned} fun_{BMA}(p_1,p_2)&= \frac{1}{2}(\frac{1}{|N_1|}\sum \nolimits _{n_1\in N_1} IC(\{n_1,n_2\})+\frac{1}{|N_2|}\sum \nolimits _{n_2\in N_2}IC(\{n_1,n_2\})) \end{aligned}$$

Onto2Vec [36] mainly employed the word2vec model [34] together with the skip-gram method to learn from the corpus derived from descriptive axioms of GO terms and proteins. For a word sequence *W* that is composed of $w_1$, $w_2,\ldots , w_S$, the skip-gram algorithm maximizes the average log-likelihood of the loss function, given by Eq. (18),

18 $$\begin{aligned} loss = \frac{1}{S} \sum _{s=1}^{S}\sum _{-|W|\le i \le |W|, i\ne 0} \log p(w_{t+i}|w_t) \end{aligned}$$

where |*W*| represents the size of training text while *S* represents the size of the vocabulary. After learning the word vectors through the word2vec model, Onto2Vec linearly combines these learned word vectors for proteins based on these words that appear in the descriptive axioms of proteins

19 $$\begin{aligned} {\mathbf {v}}(p)=\sum _{w_i\in W} {\mathbf {v}}(w_i) \end{aligned}$$

where ${\mathbf {v}}(p)$ represents the vector of protein *p*, ${\mathbf {v}}(w_i)$ represents the vector of word $w_i$, and *W* represents the set of words that appear in the descriptive axiom of protein *p*.

### Evaluation metrics

The performances of missing and spurious PPI predictions are evaluated according to the metric of area under the ROC (Receiver Operating Characteristic) curve (AUC). AUC-ROC has been widely used to evaluate the tasks of classification and prediction. ROC is calculated according to the relationship between the rate of true positives (RTP) and the rate of false positives (RFP). RTP is calculated by $RTP=\frac{TP}{TP+FN}$ and RFP is calculated by $RFP=\frac{FP}{FP+TN}$, where *TP* represents the number of true positives, while *FP* represent the number of false positives; *TN* represents the number of true negatives, while *FN* represents the number of false negatives. Tables 7 and 8 illustrate the setting of true-positive, false-positive, true-negative, and false-negative cases for the tasks of missing and spurious PPI predictions.

**Table 7:** Setting of true-positive, false-positive, true-negative, and false negative cases for missing PPIs

	Actual	
	Missing PPI	Non-miss PPI
Predicted		
Missing PPI	True positive	False positive
Non-Miss PPI	False negative	True negative

**Table 8:** Setting of true-positive, false-positive, true-negative, and false negative cases for spurious PPIs

	Actual	
	Spurious PPI	Non-spur PPI
Predicted		
Spurious PPI	True positive	False positive
Non-Spur PPI	False negative	True negative

## Data Availability

The datasets that are used in this paper can be found from their links. Gene Ontology (date of visit: 23 June 2018): http://geneontology.org/docs/download-ontology/. Gene Ontology annotations (date of visit: 23 June 2018): https://www.uniprot.org/. Protein–protein interaction datasets (date of visit: 30 October 2018): https://string-db.org/cgi/input.pl.

## Abbreviations

GO: Gene ontology
GOA: Gene ontology annotations
BP: Biological process
CC: Cellular component
MF: Molecular function
IC: Information content
PPI: Protein–protein interaction
PPIN: Protein–protein interaction network
MHD: Modified Hausdorff distance
SVM: Support vector machine
ROC: Receiver operating characteristic
AUC: Area under the curve

## Footnotes

1 https://www.uniprot.org/.

2 https://github.com/aditya-grover/node2vec.
